# Effects of *Phyllanthus amarus* PHYLLPRO^TM ^leaves on hangover symptoms: a randomized, double-blind, placebo-controlled crossover study

**DOI:** 10.1080/13880209.2019.1585460

**Published:** 2019-03-28

**Authors:** Annie George, Jay K. Udani, Ashril Yusof

**Affiliations:** a Institute of Biological Sciences, Faculty of Science, University of Malaya, Kuala Lumpur, Malaysia;; b Biotropics Malaysia Berhad, Selangor, Malaysia;; c Medicus Research LLC, Northridge, CA, USA;; d Centre for Sports and Exercise Sciences, Exercise Science, University of Malaya, Kuala Lumpur, Malaysia

**Keywords:** Antioxidant, inflammation, immunomodulatory, intoxication, alcohol, interleukin, clinical

## Abstract

**Context:** *Phyllanthus amarus* Schumach. and Thonn. (Euphorbiaceae) is traditionally known to improve general liver health. However, its effect on hangover is unknown.

**Objective:** This study evaluated PHYLLPRO™, a standardized ethanol extract of *P. amarus* leaves for protection against oxidative stress and recovery from hangover symptoms.

**Material and methods:** Ten days daily oral supplementation of 750 mg/day followed by intoxication was evaluated in a randomized placebo-controlled (containing only excipient), crossover study in 15 subjects (21–50 years old), for oxidative stress, liver damage, alleviating hangover symptoms (Hangover Severity Score: HSS) and mood improvement (Profile-of-Mood-Scores: POMS).

**Results:** PHYLLPRO™ was able to remove blood alcohol in the active group while the placebo group still had 0.05% at 12 h post-intoxication (*p* < 0.0001). For HSS, the active group showed reduced hangover symptoms while there were higher levels of nausea, headache, anorexia, tremulousness, diarrhoea and dizziness in the placebo group (*p* < 0.05) at hour 10 post-intoxication. Increased fatigue at hour 2 and tension (*p* > 0.05) from baseline to hour 22 was reported in the placebo group using POMS. Significant anti-inflammatory group effect favouring the active group, by the upregulation of cytokines IL-8 (*p* = 0.0014) and IL-10 (*p* = 0.0492) and immunomodulatory effects via IL-12p70 (*p* = 0.0304) were observed. The incidence of adverse events was similar between groups indicating the safety of PHYLLPRO™.

**Discussion and conclusion:** Preliminary findings of PHYLLPRO™ in managing hangover, inflammation and liver functions following intoxication, is demonstrated. Future studies on PHYLLPRO™ in protecting against oxidative stress and hangover in larger populations is warranted.

## Introduction

The World Health Organization (WHO) estimates that the average alcohol consumption per capita in adults is 5.1 L per year based on the report by the National Institute on Alcohol Abuse and Alcoholism (Roa 2004). Alcohol dependency affects millions of individuals around the world and is associated with several types of diseases. Based on the International Classification of Disease 10th edition; ICD-10 (WHO 2018), cause of death for liver cirrhosis is associated with alcohol consumption. Although moderate alcohol intake may have some health benefits, especially in relation to cardiovascular disease, elevated intake causes hepatic damage. Alcoholic steatosis (reversible) and alcoholic steatohepatitis are stages leading to liver disease where hepatic stellate cells are activated, depositing collagen in the liver. Binge drinking, defined by the National Institute on Alcohol Abuse and Alcoholism as a pattern of drinking that brings the blood alcohol level to 0.08 g % or higher, occurs when five (for men) or four (for women) or more alcoholic beverages are consumed over a period of 2 h (Roa 2004). Health concerns associated with binge drinking include injuries, alcohol poisoning, neurological damage and liver disease. Hangovers are the body’s reaction to poisoning and withdrawal from alcohol.

Several herbal products have been studied for liver health. These include, but are not limited to, milk thistle, *Astragalus membranaceus* Bunge (Leguminosae) and dandelion (Chen 1990; Tan et al. 2001; Rambaldi et al. 2007). *Phyllanthus amarus* Schumach. and Thonn. (Euphorbiaceae) is a tropical plant commonly found in India and Indonesia, and is traditionally used for the treatment of hepatitis and jaundice, and for general liver health (Adeneye and Benebo 2008; Nworu et al. 2010; Patel et al. 2010), among other uses. Constituents of *P. amarus* include lignans, phyllanthin, hypophyllanthin phyltetralin and niranthin (Patel et al. 2011; Jantan et al. 2014).

In patients with chronic viral hepatitis B, *P. amarus* normalized alanine aminotransferase (ALT) and the albumin:globulin ratio, and the effective rate was not significantly different from patients treated with interferon (Xin-Hua et al. 2001). In addition, *P. amarus* treated group showed a decrease in ALT and bilirubin, while an increase in haemoglobin (Patel et al. 2010). Meanwhile, in animals, *P. amarus* exhibited protective effect against carbon tetrachloride-induced liver toxicity (Krithika and Verma 2009). This hepatoprotective effects of *P. amarus* is proposed to be due to its antioxidant activities (Harish and Shivanandappa 2006; Chatterjee & Sil 2006; Londhe et al. 2008; Manjrekar et al. 2008; Karuna et al. 2009) and/or its ability to normalize liver enzymes (Borah et al. 2004; Igwe et al. 2007; Vijayanand et al. 2007) as shown in animal models. While in ethanol treated rats, aqueous extract of *P. amarus* is able to reduce the AST and ALT to normal values (Pramyothin et al. 2007), while methanol extract of *P. amarus* is able to restore glutathione (GSH) levels, reduce lipid peroxidation (Faremi et al. 2008) and protect against hepatic fibrosis (Narayanan et al. 2011). However, the role of *P. amarus* in hepatoprotection against hangover symptoms and alcohol-induced injury has not been studied clinically. In the current study, we tested the hypothesis that an ethanol extract preparation of *P. amarus* leaves, PHYLLPRO™, previously associated with antioxidant and free radical scavenging activity, would protect the liver against oxidative stress induced by alcohol consumption, thus reducing hangover symptoms.

## Material and methods

### Plant material

*Phyllanthus amarus* was identified by Mr. Shamsul Khamis the taxonomist from the Institute of Bioscience, University Putra Malaysia (UPM) based on its exomorphic characteristics (voucher specimen SK3185/17). The leaves of the plant were used by Biotropics Malaysia Berhad to produce a standardized extract trademarked as PHYLLPRO™ for the clinical studies.

### Plant extract

The standardized ethanol extract of *P. amarus* trademarked as PHYLLPRO™, was determined by high performance liquid chromatography (HPLC) to contain not less than 0.1% w/w hypophyllanthin and 0.4% w/w of phyllanthin. The total phenolic content (TPC) was not more than 100 mg GAE/g dry extract. The extract was prepared by air-drying the leaves of *P. amarus*, before grinding and extraction with a mixture of ethanol and water at a ratio of 1:1 for 4 h at 45 °C. The resulting liquid was filtered and evaporated at 60–80 °C, and spray dried.

### Investigational product

The investigational product was a tablet containing 375 mg of PHYLLPRO™, the propriety standardized ethanol extract of *P. amarus* and microcrystalline cellulose, calcium phosphate dibasic dehydrate, sodium starch glycolate, glyserylbehenate and colloidal silicon dioxide as excipient, to be taken orally twice a day for 10 days. The placebo contained only the microcrystalline cellulose, calcium phosphate dibasic dehydrate, sodium starch glycolate, glyserylbehenate and colloidal silicon dioxide, at the same dosage regimen. The investigational product was produced under good manufacturing practices (GMP) requirements by Unison Nutraceutical Sdn Bhd.

### Subjects

Sixteen healthy male and female subjects between the ages of 21 and 50 with a minimum profile of mood states (POMS) score of 15 and who consumed at least five servings of alcohol per week as inclusion criteria were recruited. Subjects were asked to maintain their regular lifestyle and to not make significant changes to their diet. Exclusion criteria included any chronic liver condition, hepatitis B and C; liver function test results greater than three times the upper level limit of normal; a history of aggressive behaviour and alcoholism; the presence of ascites; any significant gastrointestinal conditions; renal, hepatic, endocrine (including diabetes mellitus), cardiac, pulmonary, pancreatic, neurologic or biliary disorders; known allergy or sensitivity to any ingredients in the study products; uncontrolled hypertension (systolic blood pressure ≥160 mmHg or diastolic blood pressure ≥100 mmHg at visit 1, week-1); a history of cancer; recent use of antibiotics; females who were pregnant, lactating or planning to become pregnant during the study period; a recent history of (within 12 months) or strong potential for alcohol or substance abuse (defined as >14 drinks per week; one drink =12 oz. of beer, 5 oz. of wine, or 1.5 oz. of distilled spirits); enrollment in any other clinical trial within 3 months prior to recruitment and investigator’s determination of unsuitability for trial participation.

### Study design

This was a single-centre, double-blind, placebo-controlled, randomized crossover study. The research was expected to generate pilot data from which future size calculations could be made. Ethics approval was obtained from Copernicus Group Institutional Review Board (IRB), Cary, NC prior to the study. All subjects signed informed consent before undergoing screening procedures. Subjects, clinical staff, data management staff and statistical analysis staff were blinded to the study group. The study was conducted in 2010 at the Staywell Research clinical research site located in Northridge, CA. The recruitment and follow-up took place from 13 July 2010 to 16 October 2010. The study was conducted in accordance with the FDA Code of Federal Regulations (CFR 312.50 and 312.56), ICH Guidelines for Good Clinical Practice: Consolidated Guidance (GCP E-6, April 1996), the Declaration of Helsinski and HIPAA (Health Insurance Portability and Accountability Act). The clinical study has been registered with clinicaltrials.gov (NCT03349697).

Patients were randomly assigned to order of treatment (placebo or active) using simple randomization based on the atmospheric noise method and sequential assignment was used to determine group allocation (GraphPad Prism 6, San Diego, CA, USA). A computer-generated list of random numbers was used to allocate participants. Initially, 10 subjects were randomized to the active group and five to the placebo group. In the second arm, these allocations were reversed. Allocation, enrollment and assignment of participants to trial product were performed by the staff of Staywell Research Centre, who did not perform any analyses or clinical procedures. Allocation was disclosed to the investigator, subjects, and a statistician after all measurements were completed. The investigational product was stored in a Study Product Container in a locked cabinet with limited access.

**Figure 1.**
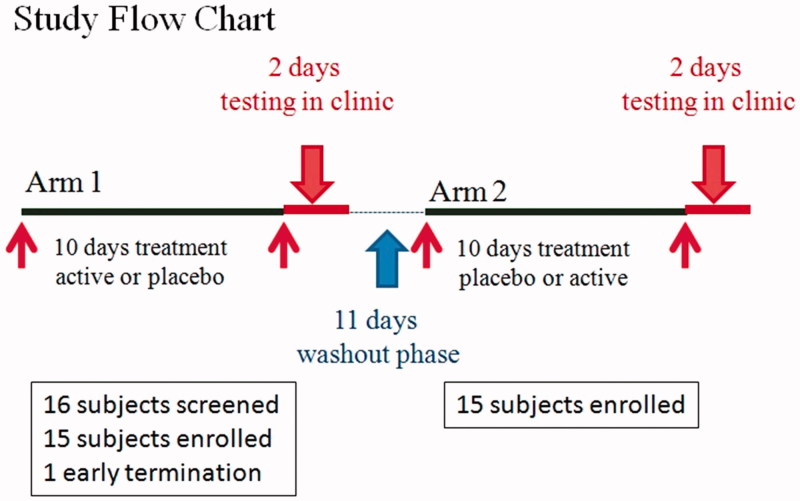
Study flow chart. Fifteen of the 16 subjects screened were randomized in the study with 15 in the PHYLLPRO™ and placebo group, crossed over and included as the per-protocol final analysis. Subjects who received PHYLLPRO™ during Arm 1 received placebo during Arm 2. Subjects who received placebo during Arm 1 received PHYLLPRO™ during Arm 2.

Subjects were required to attend a total of five visits (Figure 1). At the first visit of baseline screening, and fulfilling all inclusion and exclusion criteria, subjects were recruited and randomized before receiving standardized frozen foods (ready-to-eat after microwaving or oven-heated foods such as pizza) to consume for 36 h prior to their next visit and 10 days’ worth of study products. Subjects were instructed to avoid alcohol while taking the product and were given subject diaries to record product compliance and track caffeine intake on a daily basis.

At visit 2, after eating only the standardized foods in the 36 h prior, subjects were given several shots (1.5 fluid oz.) of alcohol (Jack Daniel’s Whisky, TN, USA) to reach a blood alcohol level (BAL) of 0.12% to create a high enough level of impairment to result in positive hangover scores the following morning (visit 3), measured using a breathanalyzer (AlcoMate® PRO, Korea). Whisky was chosen for its high congener content relative to other types of alcohol (Wiese et al. 2004). The number of shots was estimated based on gender and body weight. Breath analyzer measurements were taken at 30 and 60 min after the initiation of alcohol ingestion. During the overnight stay, the primary, secondary and tertiary endpoints were measured over 22 h upon intoxication. At the end of the visit, subjects received standardized frozen foods to consume for 36 h prior to switching to the next arm in the next visit (visit 4).

Subjects then underwent an 11-day washout period and were crossed over to receive the other arm and re-assessed. Visits 4 and 5 were the same as visits 2 and 3, until all subjects completed the trial.

Subjects were allowed to discontinue from the study if serious pathological findings occurred, which would require follow-up until resolution or until the findings were fully clarified or reasonably explained. Study data of discontinued subjects would be fully documented up to the time of their withdrawal. If an adverse event was the reason for discontinuation, the event was followed up and documented as per protocol. In case of withdrawal, the investigator would indicate in the Case Report Form one primary reason for discontinuation and as many secondary reasons as were applicable

### Outcome measurements

Primary endpoints were C-reactive protein (CRP), an inflammatory cytokine panel (tumour necrosis factor (TNF)-α, interleukin (IL)-1β, IL-2, IL-4, IL-5, IL-6, IL-7, IL-8, IL-10, IL-12p70, IL-13, IL-17A, granulocyte [G]-colony stimulating factor [CSF], eotaxin, human macrophage inflammatory protein [MIP-1β], interferon (IFN)-γ, vascular endothelial growth factor (VEGF), monocyte chemotactic protein (MCP)-1, fibroblast growth factor (FGF)-BASIC, and granulocyte macrophage (GM-CSF) analyzed by Affymetrix (San Diego, CA), liver function tests and glutathione peroxidase [GSH-Px] analyzed by American Analytical Chemistry Laboratories Corp. (Champaign, IL). Blood alcohol levels were assessed by breathanalyzer. The secondary endpoints were the Hangover Severity Score and POMS. The Hangover Severity Score is a unit of measurement on a seven-point scale from 0 to 6. Healthy subjects should have a score of 0. Symptoms on the scale include nausea, headache, lack of appetite, dry mouth, soreness, weakness, tremulousness, diarrhoea and dizziness (Wiese et al. 2004). Two additional questions were added to assess the overall feeling of the subject at that time point and their ability to attend work or school if applicable.

POMS is a psychological rating scale used to assess transient, distinct mood states (McNair et al. 1992). The moods included are tension, depression, anger, vigour, fatigue, confusion and total mood disturbance.

The tertiary endpoints were safety measured by complete blood count with differential electrical resistance methods, comprehensive metabolic panel using the spectrophotometric method, urinalysis outsourced to Primex Laboratories, Cary, NC, electrocardiogram analysis and adverse events.

### Statistical analysis

A per-protocol sample was used for this analysis. Only patients who completed the entire clinical trial were counted towards the final results and included the 15 subjects. Statistical analyses, group × time interaction for cytokine panel, blood alcohol levels, Hangover Severity Score and POMS were performed using a two-way repeated ANOVA. Shapiro–Wilk normality testing was carried out to determine data normality where *p* > 0.05 was found. Within-group comparisons were made using a paired *t*-test. Student’s *t*-test was used in the analysis of hepatoprotection assay. All statistical analyses (descriptive statistics and Student’s *t*-tests) were performed using SPSS Base System Ver. 18 (IBM SPSS Inc., Chicago, IL). The level of significance was set at *p* < 0.05.

## Results

### Baseline characteristics of subjects

The baseline characteristics of the 15 subjects are provided in Table 1. Sixteen subjects were screened in this study. One terminated early because of the inability to tolerate the amount of alcohol required to reach the target BAL. Fifteen subjects completed both arms of the study (Figure 1). There were no differences in compliance between the groups (95.8% in both groups).

**Table 1.** Baseline characteristics of subjects.

Baseline characteristics	
*N*	15
Male number (percentage)	10 (66.67%)
Female number (percentage)	5 (33.33%)
Age in years (mean)	25 ± 8.9
Age in years (range)	21–47
Weight in lbs. (mean)	155.6 ± 15.1
Weight in lbs. (range)	134–193
BMI (mean)	23.9 ± 2.1
BMI (range) kg/m^2^	20.4–28.3

### Blood alcohol levels

Females in both treatment groups consumed five shots of alcohol each. Males consumed 6–8 shots of alcohol, with an equivalent number of shots consumed during both treatment phases. BAL was consistent with intoxication by hour 0.5. The groups showed no difference until hour 12, when BAL in the active group had returned to zero, while the placebo group still had BAL of 0.05% (*p* < 0.0001; *F* = 12.73) (Table 2).

**Table 2.** Effect of treatment on blood alcohol level (mean ± SD).

	Baseline	0.5 h	1 h	1.5 h	12 h	18 h	22 h
Active	0.000 ± 0.000	0.110 ± 0.021	0.130 ± 0.022	0.130 ± 0.014	0.000 ± 0.018^a^	0.000 ± 0.000	0.000 ± 0.000
Placebo	0.000 ± 0.000	0.101 ± 0.032	0.120 ± 0.025	0.140 ± 0.016	0.050 ± 0.120	0.000 ± 0.000	0.000 ± 0.000

^a^Denotes significant difference between groups *p* < 0.0001.

### Anti-inflammatory effects

There were no statistical differences in inflammatory cytokines between the groups at any time point except for IL-8 (*p* = 0.0014), IL-10 (*p* = 0.0492) and IL-12p70 (*p* = 0.0304), where significant increases in the active group and decreases in the placebo group were observed (Table 3). In the liver function tests, no significant differences were observed between the groups.

**Table 3.** Inflammatory cytokine panel and liver function of subjects at baseline to 22 h.

	Baseline		Hour 2		Hour 12		Hour 22	
	treatment	Placebo	treatment	Placebo	treatment	Placebo	treatment	Placebo
CRP	0.89 ± 0.97	0.72 ± 0.88	0.90 ± 0.92	0.72 ± 0.82	0.83 ± 0.72	0.68 ± 0.79	0.86 ± 0.87	0.73 ± 0.85
Inflammatory cytokine panel								
TNF-α	4.74 ± 1.10	5.07 ± 1.39	4.74 ± 1.03	5.00 ± 1.25	4.25 ± 1.14	4.52 ± 0.74	5.16 ± 1.68	5.30 ± 1.16
IL-1β	8.27 ± 3.73	7.81 ± 1.88	7.32 ± 1.79	7.82 ± 1.84	7.48 ± 1.79	7.60 ± 2.09	8.47 ± 1.92	8.40 ± 2.73
IL-2	4.17 ± 0.65	4.48 ± 0.83	4.15 ± 0.51	4.23 ± 0.84	4.01 ± 0.65	3.96 ± 0.64	4.43 ± 0.91	4.71 ± 1.14
IL-4	5.39 ± 1.60	5.09 ± 1.10	5.08 ± 1.56	5.27 ± 1.44	4.76 ± 1.57	4.79 + 0.63	5.92 ± 2.26	5.21 ± 1.07
IL-5	4.36 ± 1.55	4.16 ± 0.98	405 ± 1.07	4.67 ± 1.05	3.90 ± 1.00	4.45 ± 1.79	4.15 ± 0.94	4.70 ± 1.75
IL-6	2.94 ± 0.26	3.02 ± 0.05	3.02 ± 0.05	3.02 ± 0.05	3.00 ± 0.38	2.97 ± 0.13	3.27 ± 0.46	3.06 ± 0.14
IL-8	4.83 ± 0.79	5.24 ± 1.07	4.68 ± 0.73	4.77 ± 0.78	8.72 ± 4.84	5.69 ± 1.40	5.28 ± 0.68	4.19 ± 0.81#
IL-10	3.82 ± 0.61	4.35 ± 0.76	4.15 ± 0.62	4.04 ± 0.56	3.92 ± 0.40	4.10 ± 0.71	4.09 ± 0.45	4.23 ± 0.71#
IL-12p70	3.98 ± 0.80	4.99 ± 1.49	4.30 ± 0.70	4.48 ± 0.65	4.02 ± 0.54	4.41 ± 0.96	4.54 ± 0.84	4.62 ± 0.64#
IL-13	4.88 ± 0.94	4.84 ± 0.88	4.90 ± 0.96	5.21 ± 1.03	4.70 ± 0.74	4.61 ± 0.82	4.84 ± 0.84	5.11 ± 0.99
IL-17A	3.61 ± 0.47	3.87 ± 0.74	3.63 ± 0.48	3.73 ± 0.46	4.00 ± 0.53	3.68 ± 0.45	3.53 ± 0.52	3.67 ± 0.49
GM-CSF factor	5.87 ± 0.99	5.84 ± 0.53	5.55 ± 0.48	6.24 ± 0.78	5.51 ± 0.63	5.85 ± 0.80	6.27 ± 1.16	6.23 ± 1.01
Eotaxin	54.10 ± 39.89	68.67 ± 44.12	61.07 ± 50.93	55.02 ± 30.01	90.33 ± 61.2	92.93 ± 62.66	98.57 ± 67.84	87 ± 61.56
MIP-1beta	422.67 ± 161.14	410.72 ± 171.99	411.07 ± 195.48	381.52 ± 176.67	556.52 ± 183.08	586.23 ± 222.62	462.71 ± 145.04	485.18 ± 197.25
IFN-γ	5.67 ± 1.77	6.40 ± 2.10	5.66 ± 1.63	5.49 ± 1.26	5.54 ± 2.04	6.03 ± 2.62	5.78 ± 1.91	6.18 ± 2.32
VEGF	47.86 ± 24.02	61.67 ± 40.74	45.65 ± 24.23	51.33 ± 30.81	57.64 ± 37.68	64.72 ± 44.88	54.89 ± 29.34	62.12 ± 37.67
MCP-1	16.43 ± 6.31	15.54 ± 4.66	14.35 ± 5.09	15.15 ± 4.66	18.28 ± 5.21	20.64 ± 8.28	16.8 ± 4.67	17.55 ± 5.80
FGF-basic	4.31 ± 0.46	4.46 ± 0.51	4.30 ± 0.59	4.50 ± 0.56	4.55 ± 0.89	4.32 ± 0.46	4.70 ± 0.45	4.51 ± 0.50
Liver function tests								
AST	21.8 ± 8.61	18.13 ± 5.30	21.6 ± 8.26	18.87 ± 5.14	18.79 ± 6.58	17.07 ± 3.9	17.08 ± 4.91	17.27 ± 3.84
ALT	18.13 ± 8.79	16 ± 5.68	18.33 ± 8.74	16.13 ± 5.65	16.21 ± 8.38	15.4 ± 5.15	15.92 ± 8.25	15.53 ± 5.64
Total bilirubin	0.51 ± 0.2	0.49 ± 0.22	0.48 ± 0.15	0.47 ± 0.17	0.42 ± 0.15	0.39 ± 0.12	0.59 ± 0.21	0.54 ± 0.22
Albumin	4.53 ± 0.29	4.41 ± 0.32	4.59 ± 0.35	4.45 ± 0.34	4.36 ± 0.35	4.36 ± 0.43	4.41 ± 0.31	4.45 ± 0.41
INR	1.04 ± 0.07	1.03 ± 006	1.03 ± 0.07	1.04 ± 0.06	1.06 ± 0.11	1.07 ± 0.09	1.06 ± 0.08	62.47 ± 14.88
ALK	63.88 ± 13.08	67.44 ± 14.17	66.31 ± 14.62	69.84 ± 13.31	62.84 ± 14.76	67.41 ± 15.23	62.47 ± 14.88	67.88 ± 15.61
GSH-Px	69.89 ± 15.77	74.69 ± 16.18	70.46 ± 15.52	71.48 ± 14.27	70.79 ± 9.60	75.3 ± 13.26	73.79 ± 11.66	76.86 ± 12.88

^#^ *p* < 0.05, Denotes significant difference of active compared to placebo.

### Effect of PHYLLPRO™ on hangover and mood

**Figure 2.**
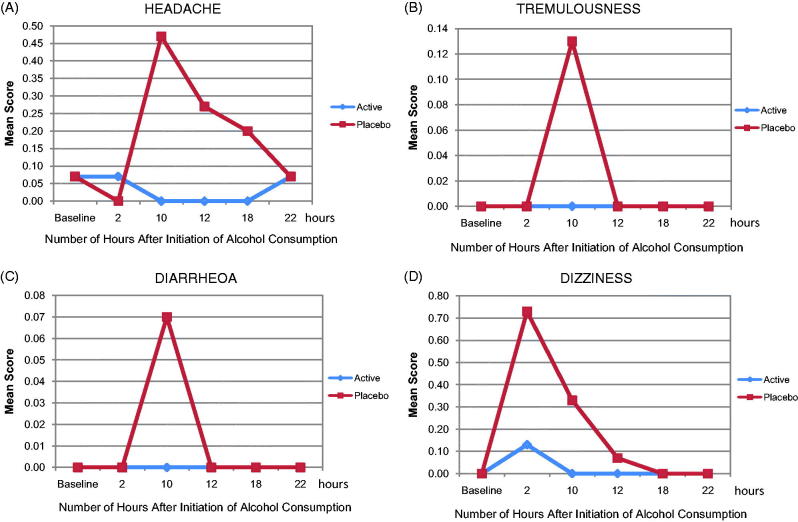
Domains of (A) headache, (B) tremulousness, (C) dizziness and (D) diarrhoea within the Hangover Severity Score in the placebo and active groups within 22 h of the initiation of alcohol.

There was no significant difference between the groups in the Hangover Severity Score. However, notable differences between the groups were observed at specific time points (Figure 2). The placebo group had higher scores than the active group (*p* < 0.05) for at least one-time point on every individual hangover symptom scale, and in some cases, the active group reported no symptoms at hours12–18 post-treatment and intoxication (Table 4).

**Table 4.** Effect of treatment on Hangover Severity Score (mean ± SD).

	Baseline		Hour 2		Hour 10		Hour 12		Hour 18		Hour 22	
	Active	Placebo	Active	Placebo	Active	Placebo	Active	Placebo	Active	Placebo	Active	Placebo
Nausea	0.00 ± 0.00	0.00 ± 0.00	0.20 ± 0.78	0.07 ± 0.26	0.00 ± 0.00	0.20 ± 0.41**	0.00 ± 0.00	0.07 ± 0.26*	0.00 ± 0.00	0.00 ± 0.00	0.00 ± 0.00	0.00 ± 0.00
Headache	0.07 ± 0.26	0.07 ± 0.26	0.07 ± 0.26	0.00 ± 0.00*	0.00 ± 0.00	0.47 ± 1.06**	0.00 ± 0.00	0.27 ± 0.79**	0.00 ± 0.00	0.20 ± 0.78*	0.07 ± 0.26	0.07 ± 0.27
Anorexia	0.00 ± 0.00	0.00 ± 0.00	0.20 ± 0.56	0.00 ± 0.00**	0.00 ± 0.00	0.20 ± 0.56**	0.13 ± 0.52	0.20 ± 0.56	0.07 ± 0.26	0.33 ± 1.29	0.00 ± 0.00	0.00 ± 0.00
Dry mouth	0.13 ± 0.35	0.00 ± 0.00	0.13 ± 0.35	0.20 ± 0.56	0.31 ± 0.75	0.40 ± 0.91	0.20 ± 0.56	0.27 ± 0.59	0.13 ± 0.35	0.07 ± 0.26	0.13 ± 0.35	0.07 ± 0.27
Soreness	0.00 ± 0.00	0.07 ± 0.26*	0.00 ± 0.00	0.00 ± 0.00	0.00 ± 0.00^a^	0.13 ± 0.35	0.07 ± 0.26	0.07 ± 0.26	0.00 ± 0.00	0.07 ± 0.26*	0.13 ± 0.52	0.07 ± 0.27*
Weakness	0.00 ± 0.00	0.07 ± 0.26*	0.00 ± 0.00	0.13 ± 0.52*	0.08 ± 0.28	0.13 ± 0.35	0.07 ± 0.26	0.07 ± 0.26	0.00 ± 0.00	0.07 ± 0.26*	0.00 ± 0.00	0.00 ± 0.00
Tremulousness	0.00 ± 0.00	0.00 ± 0.00	0.00 ± 0.00	0.00 ± 0.00	0.00 ± 0.00	0.13 ± 0.35*	0.00 ± 0.00	0.00 ± 0.00	0.00 ± 0.00	0.00 ± 0.00	0.00 ± 0.00	0.00 ± 0.00
Diarrhoea	0.00 ± 0.00	0.00 ± 0.00	0.00 ± 0.00	0.00 ± 0.00	0.00 ± 0.00	0.07 ± 0.26	0.00 ± 0.00	0.00 ± 0.00	0.00 ± 0.00	0.00 ± 0.00	0.00 ± 0.00	0.00 ± 0.00
Dizziness	0.00 ± 0.00	0.00 ± 0.00	0.13 ± 0.35	0.73 ± 1.79*	0.00 ± 0.00	0.33 ± 0.49***	0.00 ± 0.00	0.07 ± 0.26*	0.00 ± 0.00	0.00 ± 0.00	0.00 ± 0.00	0.00 ± 0.00
Overall (how do you feel right now?)	1.20 ± 1.37	1.40 ± 1.24	1.50 ± 1.45	1.20 ± 1.47	1.77 ± 1.74	2.00 ± 1.73	1.47 ± 1.35	1.67 ± 1.54	1.27 ± 1.10	1.33 ± 1.29	1.20 ± 1.21	1.07 ± 1.28
If you needed to attend work or school right now, would you be able to?	0.00 ± 0.00	0.00 ± 0.00	0.20 ± 0.41	0.20 ± 0.41	0.23 ± 0.44	0.27 ± 0.46	0.20 ± 0.41	0.07 ± 0.26*	0.00 ± 0.00	0.00 ± 0.00	0.07 ± 0.26	0.00 ± 0.00*

*Denotes significant difference *p* < 0.05, ** *p* < 0.005, *** *p* < 0.0001, compared to baseline.

a Denotes significant difference *p* < 0.05 between groups.

There were more reports of nausea in the placebo group than the active group at hours 10 (*p* = 0.04) and 12 (up to *p* < 0.001), and of headache at hours 10, 12, and 18 (*p* = 0.004; *p* = 0.009; *p* = 0.04, respectively). There were more reports of anorexia in the placebo group than the active group at hour 10 (*p* = 0.008), and of dry mouth in the placebo group at hours 10 and 12, but these differences were not statistically significant. With respect to soreness, there were more reports in the placebo group than the active group at hours 10 and 18 (*p* < 0.05). The active group reported soreness only at baseline (*p* = 0.04). There were significantly more complaints of weakness in the placebo group than in the active group at baseline, hours 2 and18 (*p* = 0.04 for both groups). There were more complaints of tremulousness in the placebo group than in the active group at hour 10 (*p* = 0.003). Results for diarrhoea domain of POMS in the placebo group were significant (*p* = 0.05) between groups at hour 10. There were significantly more complaints of dizziness in the placebo group than the active group at hours 2, 10 and 12 (*p* < 0.0001–*p* < 0.05). The scores for overall feeling were not significantly different between the groups in the population as a whole. For the ability to attend work or school if necessary, at hours 12 and 22, more subjects felt able to attend work or school in the placebo group than in the active group (*p* = 0.031).

No statistically significant differences between groups were observed in the POMS questionnaire or in its individual domains. Notable differences between time points were observed in the placebo group, which had significantly higher scores than the active group at hours 18 (*p* = 0.04) and 22 (*p* = 0.008), representing higher levels of tension at these time points (Table 5). The active group had significantly higher scores than the placebo group at hours 10 (*p* = 0.002), 12 (*p* = 0.04) and 22 (*p* = 0.04), representing higher levels of depression at these time points. The placebo group had significantly higher scores for anger than the active group at hours 2 (*p* = 0.04) and 12 (*p* = 0.026), and the active group had significantly higher scores at hour 18 (*p* < 0.0001), representing higher levels of anger at these time points. POMS vigour was not significantly different between groups at any time point. The clinically and statistically significant reduction in vigour in both groups at hour 10 can be explained by the fact that this questionnaire was administered at 4:00 am (*p* < 0.05). For fatigue, the placebo group had significantly higher scores than the active group at hour 2 (*p* = 0.033), representing higher levels of fatigue at this time point which were significantly different between the groups.

**Table 5.** The effect of treatment of Profile of Mood Scores.

	Before supplementation						After supplementation					
	Baseline		Hour 2		Hour 10		Hour 12		Hour 18		Hour 22	
	Active	Placebo	Active	Placebo	Active	Placebo	Active	Placebo	Active	Placebo	Active	Placebo
POMS												
Tension-Anxiety	0.33 ± 0.72	0.40 ± 1.06	0.07 ± 0.26	0.13 ± 0.52	0.27 ± 1.03	0.33 ± 1.05	0.00 ± 0.00	0.00 ± 0.00	0.00 ± 0.00	0.07 ± 0.26*	0.13 ± 0.52	0.67 ± 1.50*
Depression-Dejection	0.00 ± 0.00	0.00 ± 0.00	0.07 ± 0.26	0.13 ± 0.52	0.33 ± 0.82	0.00 ± 0.00*	0.07 ± 0.26	0.00 ± 0.00*	0.00 ± 0.00	0.00 ± 0.00	0.07 ± 0.26	0.00 ± 0.00*
Anger-Hostility	0.13 ± 0.52	0.020 ± 0.41	0.00 ± 0.00	0.13 ± 0.52*	0.87 ± 1.68	0.53 ± 1.36	0.13 ± 0.35	0.91 ± 0.24*	0.27 ± 0.59	0.00 ± 0.00*	0.33 ± 1.05	0.40 ± 0.91
Vigour-Activity	8.87 ± 6.65	10.40 ± 5.18	7.40 ± 5.59	9.93 ± 5.51	4.13 ± 5.14	3.80 ± 4.91	6.00 ± 6.21	6.60 ± 6.42	7.67 ± 7.08	8.47 ± 6.75	8.80 ± 7.51	9.80 ± 7.37
Fatigue-Inertia	0.73 ± 1.16	0.93 ± 1.22	0.47 ± 0.92	1.13 ± 1.81*	1.53 ± 2.07	2.20 ± 2.34	1.73 ± 2.63	1.67 ± 2.06	0.33 ± 0.72	0.60 ± 1.18	0.93 ± 2.15	0.47 ± 1.34
Confusion-Bewilderment	2.13 ± 1.99	1.60 ± 1.84	2.27 ± 1.75	2.13 ± 1.85	2.40 ± 1.72	2.67 ± 1.79	2.53 ± 1.68	2.53 ± 1.77	2.40 ± 1.99	2.13 ± 1.81	2.20 ± 1.94	1.93 ± 1.83
Overall Mood	−5.53 ± 9.97	−7.27 ± 8.10	−4.53 ± 7.32	−5.67 ± 8.25	1.27 ± 7.87	1.93 ± 8.49	−1.53 ± 8.61	−2.00 ± 9.72	−4.67 ± 9.15	−5.67 ± 9.12	−5.13 ± 11.35	−6.33 ± 10.66

*Denotes significant difference at *p* < 0.05, compared to baseline.

### Safety profile of PHYLLPRO™

The safety profile of the test product appeared to be identical to that of placebo, with no serious adverse events reported; none of the adverse events were attributed to the test product. In the active group, adverse events included mild upper respiratory infection (*n* = 2; not related) and mild headache (*n* = 1; unlikely related). In the placebo group, adverse events included vomiting (mild-to-moderate; *n* = 3; unlikely or possibly related) and mild dry mouth (*n* = 2; possibly related).

## Discussion

This clinical study provides evidence of the ability of PHYLLPRO™ to remove blood alcohol more efficiently compared with placebo, resulting in reduced hangover symptoms and improved mood. PHYLLPRO™ also demonstrated certain anti-inflammatory and antioxidant protective effect by the up-regulation of anti-inflammatory cytokine IL-8 and IL-10 upon alcohol toxification.

The main compounds isolated from *P. amarus* are lignans, flavonoids, ellagitannins, triterpenes, alkaloids, sterol and essential oil (Patel et al. 2011). The pure compounds phyllanthin and hypophyllanthin, also found in PHYLLPRO™, have high antioxidant effects with a low IC_50_ value, especially for phyllanthin with IC_50_ of 7.4 mol/mL (Sen and Batra 2013). A similar 50% ethanol extract of the aerial parts of *P. amarus* contains phyllanthin, amariin, repandusinic acid and phyllanthusiin D, flavonoids, rutin and quercetin 3-*O* glucoside, which display high antioxidant activity (Londhe et al. 2008; Krithika et al. 2009). It is well known that the TPC of an extract corresponds to its antioxidant effects (Eldeen et al. 2011). This could have contributed to the beneficial effects seen in this study.

In this study, IL-8, IL-10 and IL-12p70 levels increased significantly in the active group, denoting an anti-inflammatory effect when supplemented with PHYLLPRO™. Cytokine IL-10, a potent anti-inflammatory agent, plays a central role in preventing damage and maintaining normal tissue homeostasis (Haddad and Fahlman 2002). While IL-8, a chemokine secreted by many cell types, including monocytes, neutrophils, epithelial cells, fibroblasts, endothelial cells, mesothelial cells and tumour cells in response to inflammation (Matsushima and Oppenheim 1989), acts as a chemotaxis agent in mobilizing T cells as well as other non-specific inflammatory cells to inflammation site (Schröeder 1992). In this study, the potential liver injury during alcohol intoxication may have increased the release of IL-8 to the site of liver inflammation. Zhang et al. (2005) demonstrated that activation of the Nrf2 (NF-E2-related transcription factor 2)/antioxidant response pathway induces the expression of IL-8 to attenuate tissue injury, indicating that IL-8 responds in an anti-inflammatory manner to resolve tissue injury. The IL-12 families of cytokines are key players in the regulation of T-cell responses to infection (Gee et al. 2009) and are responsible for immune responses. Functionally, IL-12 is a heterodimeric cytokine whereby IL-12p70 is the active heterodimer with both pro- and anti-inflammatory properties. In another related study, IL-10 and IL-12 were also increased significantly compared with other cytokines during the hangover period (Kim et al. 2003) associating these increases with a response to hangover. An antioxidant-rich fermented product of fruit and vegetable caused increased levels of IL-12 accompanied by an increase in CD4+ (Zulkawi et al. 2017a) denoting an immunomodulatory response to intoxication which has been reported elsewhere (Hoek and Pastorino 2002).

Chronic ethanol intake (i.e., several years of heavy alcohol use in humans, several weeks or months in experimental animals) enhances the damaging consequences of inflammatory responses through a variety of mechanisms. It would have been expected that, in the presence of alcohol detoxification, AST would have increased as a number of liver cells may have been damaged; however, in this case, there was no significant difference in AST between the groups. Another biomarker that is commonly associated with inflammation is CRP, which in this study did not show group difference, whereas in another study, supplementation of the medicinal herb *Ginkgo biloba* L. (Ginkgoaceae) was shown to reduce CRP levels while improving total antioxidant levels in serum ischaemic stroke patients (Thanoon et al. 2012). In a study on hangover symptoms, decreased levels of CRP were associated with reduced hangover symptoms; however, liver enzymes (AST and ALT) were not reduced (Wiese et al. 2004). It is possible that the alcohol toxification in this study was not chronic enough to warrant an intense inflammatory reaction.

In this study, alcohol was consumed in a healthy population with a mean age of 25 years. It is also possible that in this young and healthy population, acute alcohol consumption prior to investigation can quickly be restored by the bodies’ normal homeostasis as the liver would be robust compared with an ageing population that has potentially been exposed to prolonged oxidative stress from various sources. This effect was observed in an animal study of young rats, where all hepatocytes entered the cell cycle after 70% liver resection, whereas only one-third did so in the aged liver (Stocker and Heine 1971), pointing towards an effect of age on liver recovery. This phenomenon could have added to the statistical non-significance between groups, which was further pronounced by the small sample size of 15 per arm.

There was a statistically significant effect of PHYLLPRO™ on POMS fatigue score and hangover symptom severity scale at several time points. The tension domain clearly demonstrated a significant increase in the placebo group and a decrease in the active group at hours 18 and 22 in the POMS. There was a significant evidence of fatigue in the placebo group at hour 2. The increase in tension and fatigue in the placebo group and not in the active group may possibly be due to antioxidative protection afforded by the extract. Oxidative stress has been linked to an increase in malondialdehyde (MDA) and psychological variables such as depression (Srivastava and Batra 2014). Oxidative stress produces higher lipid peroxidation products such as MDA in the blood than do a normal person. A significant reduction in MDA levels in CCl_4_-induced hepatotoxicity in rats was observed with the administration of *Phyllanthus niruri* L. (Phyllanthaceae), a closely related species to *P. amarus* (Karuna et al. 2009) pointing to a possible reduction in oxidative stress that would otherwise lead to higher MDA levels and poorer psychological states. Further studies are needed to confirm this for *P. amarus*.

Alcohol elimination was faster in the active group (*p* < 0.0001) at hour 12. It would have been helpful to have had additional BAL time points between hours 1.5 and 12, especially at hour 10, to evaluate the rate of alcohol detoxification since it was at hour 10 that the placebo group reported the highest complaints of nausea, headache, anorexia, tremulousness, diarrhoea and dizziness. This appears to be the time where alcohol was still present at a higher amount in the placebo group while 2 h later, the active group no longer had any alcohol in the bloodstream whereas the placebo group still did. This implies that PHYLLPRO™ improved hangover symptoms through the efficient clearing of alcohol from the bloodstream. This was the case for the fruit extract of *Evodiae rutaecarpa* (Juss.) Benth (Rutaceae) and *Xeniji™* (a trademarked fruit- and vegetable-based preparation) which reduced alcohol concentration in blood plasma *in vivo* and upregulated the relative expression of alcohol dehydrogenase (ADH) and antioxidant Cu-Zn superoxide dismutase (Cu-Zn SOD), suggesting the removal of alcohol by hepatic alcohol metabolizing and antioxidant enzymes (Cho et al. 2005; Zulkawi et al. 2017b). Other alternative therapies such as artichoke extract have been examined in a randomized controlled trial, but no benefit was found (Pittler et al. 2003). Nopal cactus [*Opuntia ficus-indica* L. Mill (Cactaceae)] was found to reduce some of the symptoms associated with hangover (Wiese et al. 2004).

The potential mechanism of action of hepatoprotection and reduction in hangover symptoms in alcohol-induced toxicity may be exerted through the improved antioxidant status as *P. amarus* demonstrated liver function improvement through decreased levels of liver function enzymes and increased antioxidant enzymes (Pramyothin et al. 2007; Faremi et al. 2008). There was an increasing trend in GSH-Px in the active group, although it was not significant, indicating increasing antioxidant activity in the blood in support of the free radical-producing damage caused by alcohol. In other *in vivo* studies, a significant increase in GSH-Px through antioxidant administration and an increase in liver enzymes suggesting liver damage through alcohol consumption as a result of chronic alcohol administration for up to 15 days was observed in untreated rats (Vogt and Richie 2007; Wang et al. 2015). In addition, the detrimental effects of alcohol were more apparent in older rats compared with younger adult rats, with the effects of oxidative stress and free radicals more prevalent. In this study, once-only acute alcohol administration in a young population (mean age of 25 years) might not have been sufficient to induce damaging effects and an intense oxidative stress reaction, as observed by the normal levels of liver enzymes, to result in a statistically significant improvement in GSH-Px following *P. amarus* supplementation.

Hepatoprotective effects of medicinal plants against short-term toxins have been observed in animal studies (Hwang et al. 2002). The detoxification effect seen particularly in the liver has been reported in several closely related *Phyllanthus* species (Karuna et al. 2011). Since clinical evidence in support of any compound for the reduction of hangover symptoms is lacking, the clinical evidence presented in this study is of interest and supports further studies in larger groups of individuals. As this was a pilot study, only 15 subjects were included. Future studies may include an appropriate number of subjects based on these preliminary findings. The effect of *P. amarus* on levels of inflammatory markers and liver enzymes following alcohol consumption as an example of inducing oxidative stress is of interest in larger populations.

## Conclusions

The ethanol extract of *P. amarus*, PHYLLPRO™, significantly improved hangover symptoms when compared with placebo. The extract also showed some immunomodulatory and anti-inflammatory effects. In addition, the product was well tolerated, with no serious adverse events observed in either the placebo or the active group at an administered dose of 375 mg twice daily for 10 days. The results of this study suggest that this product may be of interest to generally healthy adults for the treatment of hangover symptoms and for protection against oxidative stress induced by various assaults. Further research is still needed to confirm the mechanism of action of PHYLLPRO™, however, and to study its effects in older individuals and in individuals drinking varying amounts of alcohol.

## References

[CIT0001] Adeneye AA, Benebo AS 2008 Protective effect of the aqueous leaf and seed extract of *Phyllanthus amarus* on gentamicin and acetaminophen-induced nephrotoxic rats. J Ethnopharmacol. 118:318–323. 18554830 10.1016/j.jep.2008.04.025

[CIT0002] Borah RC, Mohan P, Choudhury BH, Barua IC 2004 Hepatoprotective activity of Phyllanthus fraternus L. and Glycosmis pentaphylla (Retz.) correa used against jaundice in N. E. India. Bioprospecting of commercially important plants. Proceedings of the National Symposium on Biochemical Approaches for Utilization and Exploitation of Commercially Important Plants; pp. 259–262.

[CIT0003] Chatterjee M, Sil PC 2006 Hepatoprotective effect of aqueous extract of *Phyllanthus niruri* on nimesulide-induced oxidative stress *in vivo* . Indian J Biochem Biophys. 43:299–305. 17133737

[CIT0004] Chen Z 1990 Clinical study of 96 cases with chronic hepatitis B treated with jiedu yanggan gao by a double-blind method. Zhongguo Xi Yi Jie He Za Zhi. 10:71–74. 2364464

[CIT0005] Cho MH, Shim SM, Lee SR, Mar W, Kim GH 2005 Effect of *Evodiae fructus* extracts on gene expressions related with alcohol metabolism and antioxidation in ethanol-loaded mice. Food Chem Toxicol. 43:1365–1371. 15913877 10.1016/j.fct.2005.03.010

[CIT0006] Eldeen IMS, Seow E-M, Abdullah R, Sulaiman SF 2011 *In vitro* antibacterial, antioxidant, total phenolic contents and anti-HIV-1 reverse transcriptase activities of extracts of seven *Phyllanthu*s sp. S Afr J Bot. 77:75–79.

[CIT0007] Faremi TY, Suru SM, Fafunso MA, Obioha UE 2008 Hepatoprotective potentials of *Phyllanthus amarus* against ethanol-induced oxidative stress in rats. Food Chem Toxicol. 46:2658–2664. 18524448 10.1016/j.fct.2008.04.022

[CIT0008] Gee K, Guzzo C, Che Mat NF, Ma W, Kumar A 2009 The IL-12 family of cytokines in infection, inflammation and autoimmune disorders. Inflamm Allergy Drug Targets. 8:40–52. 19275692 10.2174/187152809787582507

[CIT0009] Haddad JJ, Fahlman CS 2002 Redox- and oxidant-mediated regulation of interleukin-10: an anti-inflammatory, antioxidant cytokine? Biochem Biophys Res Commun. 297:163–176. 12237098 10.1016/s0006-291x(02)02094-6

[CIT0010] Harish R, Shivanandappa T 2006 Antioxidant activity and hepatoprotective potential of *Phyllanthus niruri* . Food Chem. 95:180–185.

[CIT0011] Hoek JB, Pastorino JG 2002 Ethanol, oxidative stress, and cytokine-induced liver cell injury. Alcohol. 27:63–68. 12062639 10.1016/s0741-8329(02)00215-x

[CIT0012] Hwang JM, Wang CJ, Chou FP, Tseng TH, Hsieh YS, Lin WL, Chu CY 2002 Inhibitory effect of berberine on *tert*-butyl hydroperoxide induced oxidative damage in rat liver. Arch Toxicology. 76:664–670. 10.1007/s00204-002-0351-9 12415430

[CIT0013] Igwe CU, Nwaogu LA, Ujuwondu CO 2007 Assessment of the hepatic effects, phytochemical and proximate compositions of *Phyllanthus amarus* . Afr J Biotechnol. 6:728–731.

[CIT0014] Jantan I, Ilangkovan M, Yuandani, Hazni FM 2014 Correlation between the major components of *Phyllanthus amarus* and *Phyllanthus urinaria* and their inhibitory effects on phagocytic activity of human neutrophils. BMC Complement Altern Med. 14:429.

[CIT0015] Karuna R, Bharathi VG, Reddy SS, Ramesh B, Saralakumari D 2011 Protective effects of *Phyllanthus amarus* aqueous extract against renal oxidative stress in Streptozotocin -induced diabetic rats . Indian J Pharmacol. 43:414–418. 21844996 10.4103/0253-7613.83112 PMC3153704

[CIT0016] Karuna R, Reddy SS, Baskar R, Saralakumari D 2009 Antioxidant potential of aqueous extract of *Phyllanthus amarus* in rats. Indian J Pharmacol. 41:64–67. 20336219 10.4103/0253-7613.51342 PMC2841234

[CIT0017] Kim DJ, Kim W, Yoon SJ, Choi BM, Kim JS, Go HJ, Kim YK, Jeong J 2003 Effects of alcohol hangover on cytokine production in healthy subjects. Alcohol. 31:167–170. 14693266 10.1016/j.alcohol.2003.09.003

[CIT0018] Krithika R, Verma RJ 2009 Mitigation of carbon tetrachloride-induced damage by *Phyllanthus amarus* in liver of mice. Acta Poloniae Pharmaceutica. 66:439–444. 19702178

[CIT0019] Krithika R, Mohankumar R, Verma RJ, Shrivastav PS, Mohamad IL, Gunasekaran P, Narasimhan S 2009 Isolation, characterization and antioxidative effect of phyllanthin against CCL_4_-induced toxicity in HEPG2 cell line. Chem Biol Interact. 181:351–358. 19576190 10.1016/j.cbi.2009.06.014

[CIT0020] Londhe JS, Devasagayam TP, Foo LY, Ghaskadbi SS 2008 Antioxidant activity of some polyphenol constituents of the medicinal plant *Phyllanthus amarus* Linn. Redox Rep. 13:199–207. 18796238 10.1179/135100008X308984

[CIT0021] Manjrekar AP, Jisha V, Bag PP, Adhikary B, Pai MM, Hegde A, Nandini M 2008 Effect of *Phyllanthus niruri* Linn. treatment on liver, kidney and testes in CCL_4_ induced hepatotoxic rats. Indian J Exp Biol. 46:514–520. 18807755

[CIT0022] Matsushima K, Oppenheim JJ 1989 Interleukin 8 and MCAF: novel inflammatory cytokines inducible by IL 1 and TNF. Cytokine. 1:2–13. 2491503 10.1016/1043-4666(89)91043-0

[CIT0023] McNair DM, Lorr M, Droppleman LF 1992 Profile of mood states manual. San Diego: Educational and Industrial Testing Service.

[CIT0024] Narayanan BS, Latha P, Rukkumani R 2011 Protective effects of *Phyllanthus amarus* on fibrotic markers during alcohol and polyunsaturated fatty acid-induced toxicity. Toxicol Mech Methods. 21:48–52. 21047178 10.3109/15376516.2010.529189

[CIT0025] Nworu CS, Akah PA, Okoye FB, Esimone CO 2010 Aqueous extract of *Phyllanthus niruri* (Euphorbiaceae) enhances the phenotypic and functional maturation of bone marrow-derived dendritic cells and their antigen-presentation function. Immunopharmacol Immunotoxicol. 32:393–401. 20095802 10.3109/08923970903463939

[CIT0026] Patel HJ, Patel JS, Patel KN, Seth AK, Patel KD 2010 Clinical study of hepatoprotective drug *Phyllanthus amarus* . Res J Pharm Biol Chem Sci. 1:335–340.

[CIT0027] Patel JR, Tripathi P, Sharma V, Chauhan NS, Dixit VK 2011 *Phyllanthus amarus*: ethnomedicinal uses, phytochemistry and pharmacology: a review. J Ethnopharmacol. 138:286–313. 21982793 10.1016/j.jep.2011.09.040

[CIT0028] Pittler MH, White AR, Stevinson C, Ernst E 2003 Effectiveness of artichoke extract in preventing alcohol-induced hangovers: a randomized controlled trial. CMAJ. 169:1269–1273. 14662662 PMC280580

[CIT0029] Pramyothin PC, Ngamtin S, Poungshompoo S, Chaichantipyuth C 2007 Hepatoprotective activity of *Phyllanthus amarus* Schum. et. Thonn. extract in ethanol treated rats: *in vitro* and *in vivo* studies. J Ethnopharmacol. 114:169–173. 17870264 10.1016/j.jep.2007.07.037

[CIT0030] Rambaldi A, Jacobs BP, Gluud C 2007 Milk thistle for alcoholic and/or hepatitis B or C virus liver diseases. Cochrane Database Syst Rev. 4:CD003620. 10.1002/14651858.CD003620.pub3 PMC8724782 17943794

[CIT0031] Roa G 2004 Featuring information from the national institute on alcohol abuse and alcoholism. NIAAA Newsletter. 3:3.

[CIT0032] Schröder JM 1992 The neutrophil-activating peptide 1/interleukin 8, a novel neutrophil chemotactic cytokine. Archivum Immunologiae et Therapia Experimentalis. 40:23–31. 1485824

[CIT0033] Sen A, Batra A 2013 The study of *in vitro* and *in vivo* antioxidant activity and total phenolic content of *Phyllanthus amarus* Schum Thonn: a medicinally important plant. Int J Pharm Pharmaceutical Sci. 5:942–947.

[CIT0034] Srivastava R, Batra J 2014 Oxidative stress and psychological functioning among medical students. Ind Psychiatry J. 23:127–133. 25788802 10.4103/0972-6748.151684 PMC4361975

[CIT0035] Stocker E, Heine WD 1971 Regeneration of liver parenchyma under normal and pathological conditions. Beitrage Zur Pathologie. 144:400–408. 5158468

[CIT0036] Tan YW, Yin YM, Yu XJ 2001 Influence of *Salvia miltiorrhizae* and *Astragalus membranaceus* on hemodynamics and liver fibrosis indexes in liver cirrhotic patients with portal hypertension. Zhongguo Zhong Xi Yi Jie He Za Zhi. 21:351–353. 12577420

[CIT0037] Thanoon IAJ, Abdul-Jabbar HAS, Taha DA 2012 Oxidative stress and C-reactive protein in patients with cerebrovascular accident (Ischaemic Stroke): the role of *Ginkgo biloba* extract. Sultan Qaboos Univ Med J. 12:197–205. 22548139 10.12816/0003113 PMC3327567

[CIT0038] Vijayanand V, Nagarajan B, Dhanapalan P 2007 Efficacy of *Azadirachta indica* and *Phyllanthus amarus* in experimental hepatitis in dogs. Indian Vet J. 85:599–602.

[CIT0039] Vogt BL, Richie JP 2007 Glutathione depletion and recovery after acute ethanol administration in the aging mouse. Biochem Pharmacol. 73:1613–1621. 17343832 10.1016/j.bcp.2007.01.033 PMC1930162

[CIT0040] Wang J, Zhang Y, Liu R, Li X, Cui Y, Qu L 2015 Geniposide protects against acute alcohol-induced liver injury in mice via up-regulating the expression of the main antioxidant enzymes. Can J Physiol Pharmacol. 93:261–267. 25730420 10.1139/cjpp-2014-0536

[CIT0041] Wiese JS, McPherson S, Odden MC, Shlipak MG 2004 Effect of *Opuntia ficus indica* on symptoms of the alcohol hangover. Archiv Internal Med. 164:1334–1340. 10.1001/archinte.164.12.1334 15226168

[CIT0042] World Health Organization (WHO). 2018 International Classification of Disease. 10th Edition. [accessed 2018 June 18]. Available from: http://www.who.int/classifications/icd/en/

[CIT0043] Xin-Hua W, Chang-Qin L, Xing-Bo G, Lin-Chun F 2001 A comparative study of *Phyllanthus amarus* compound and interferon in the treatment of chronic viral hepatitis B. Southeast Asian J Trop Med Public Health. 32:140–142. 11485076

[CIT0044] Zhang X, Chen X, Song H, Chen HZ, Rovin BH 2005 Activation of the Nrf2/antioxidant response pathway increases IL-8 expression. Eur J Immunol. 35:3258–3267. 16220540 10.1002/eji.200526116

[CIT0045] Zulkawi N, Ng KH, Zamberi R, Yeap SK, Satharasinghe D, Jaganath IB, Jamaluddin AB, Tan SW, Ho WY, Alitheen NB, et al. 2017a *In vitro* characterization and *in vivo* toxicity, antioxidant and immunomodulatory effect of fermented foods; Xeniji™. BMC Complement Altern Med. 17:344. 28666436 10.1186/s12906-017-1845-6 PMC5493119

[CIT0046] Zulkawi N, Ng KH, Zamberi R, Yeap SK, Jaganath IB, Satharasinghe D, Yong CH, Jamaluddin A, Tan SW, Ho WY, et al. 2017b The *in vivo* hepato-recovery effects of the polyphenol-rich fermented food Xeniji™ on ethanol-induced liver damage. RSC Adv. 7:38287–38299.

